# Mucoacinar Carcinoma of the Parotid Gland

**DOI:** 10.1007/s12105-026-01889-0

**Published:** 2026-02-04

**Authors:** Rachel Chang, Alexander Mackinnon, Benjamin J. Greene, Melad N. Dababneh

**Affiliations:** 1 Department of Pathology, Heersink School of Medicine, University of Alabama at Birmingham, NP3552, 619 19th St S, Birmingham, AL 35249 USA; 2 Department of Otolaryngology, Head and Neck Surgery, Heersink School of Medicine, University of Alabama at Birmingham, Birmingham, AL USA

**Keywords:** Mucoacinar carcinoma, Mucoepidermoid carcinoma, Acinic differentiation, MAML2

## Abstract

Mucoacinar carcinoma is an exceedingly rare subtype of mucoepidermoid carcinoma with serous acinar differentiation. We present this case to contribute to the limited existing literature, with emphasis on its molecular profile and unique histomorphology and immunophenotype.

Mucoepidermoid carcinoma (MEC) is the most common salivary gland malignancy, displaying recurrent *MAML2* gene rearrangement. Multiple histomorphologic presentations and subtypes have been described, including clear, sclerosing, ciliated, oncocytic, Warthin-like, and lacking detectable squamoid/squamous differentiation [1–3]. Mucoacinar carcinoma refers to a recently described rare MEC subtype displaying serous acinar differentiation, with only 12 cases reported to date [4, 5]. Distinguishing it from acinic cell carcinoma can be challenging, particularly with the emergence of the phenotypically comparable squamoglandular subtype [6].

This is a case of a 66-year-old female patient with a slowly enlarging parotid gland mass. Imaging revealed a solid-cystic mass, measuring < 2.0 cm in greatest dimension. Fine needle aspiration was attempted but the cytology specimens were comprised of cyst content only. The lesion was subsequently and completely excised, and histologic examination revealed a predominantly cystic salivary proliferation with scattered squamoglandular and intermediate cell components, surrounded focally by tumor-associated lymphoid proliferation. Variable serous acinar differentiation (the granules additionally highlighted by PAS-D special stain) and rare luminal intracytoplasmic mucin (highlighted by Mucicarmine special stain) were identified (Fig. 1). Importantly, the components were intimately admixed in a single nodule and did not display geographically separate and morphologically distinct cellular proliferations, arguing against a collision of two tumors [7]. No infiltrative growth, perineural or angiolymphatic invasion, marked cytologic atypia, increased mitoses or tumor necrosis were identified, corresponding to a low-grade categorization per the Brandwein, MSK and modified MSK MEC grading systems [8].

**Fig. 1:**
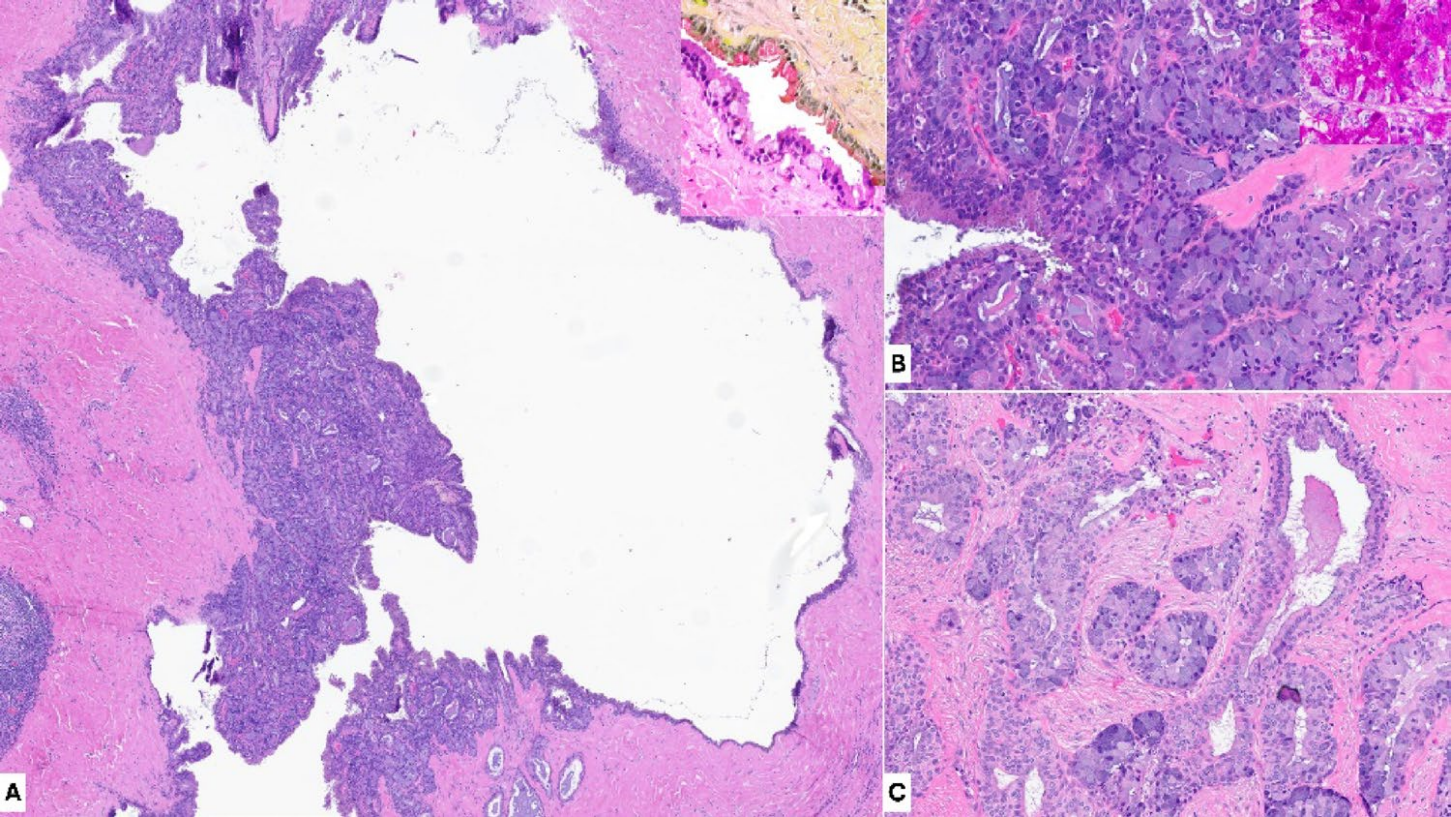
Mucoacinar carcinoma has cystic architecture with variable complex basaloid glandular proliferation (A, H&E). The cystic columnar epithelial lining shows focal intracytoplasmic mucin (**A** inset, H&E and Mucicarmine stain). Multifocal serous acinar differentiation with intracytoplasmic granules were present (**B** inset is PAS-D stain), intermingled with granules-poor squamoglandular component (**C**)

By immunohistochemistry, the lesional cells showed positive expression for CK7 (diffuse), CK5/6 (diffuse and accentuated basally/peripherally), SOX10 (variable, focally strong in the acinar component), S100 (variable, overall patchy), and DOG1 (luminal staining in the acinar component). p40 highlighted basal/abluminal cells in the serous acinar units and the simple cystic component but showed more “parabasal” staining in the complex squamoglandular areas sparing the luminal/ductal cells. Figure 2. A custom solid tumor gene fusion next-generation sequencing panel (ArcherDX FusionPlex) identified a sole *CRTC1::MAML2* fusion, without *NR4A3* gene rearrangement. No extended transcriptomic analysis was performed. The molecular results argue against a collision tumor, and the collective evidence is consistent with a MEC subtype. No further management was indicated, and recent post-operative follow-up data is not available.

**Fig. 2:**
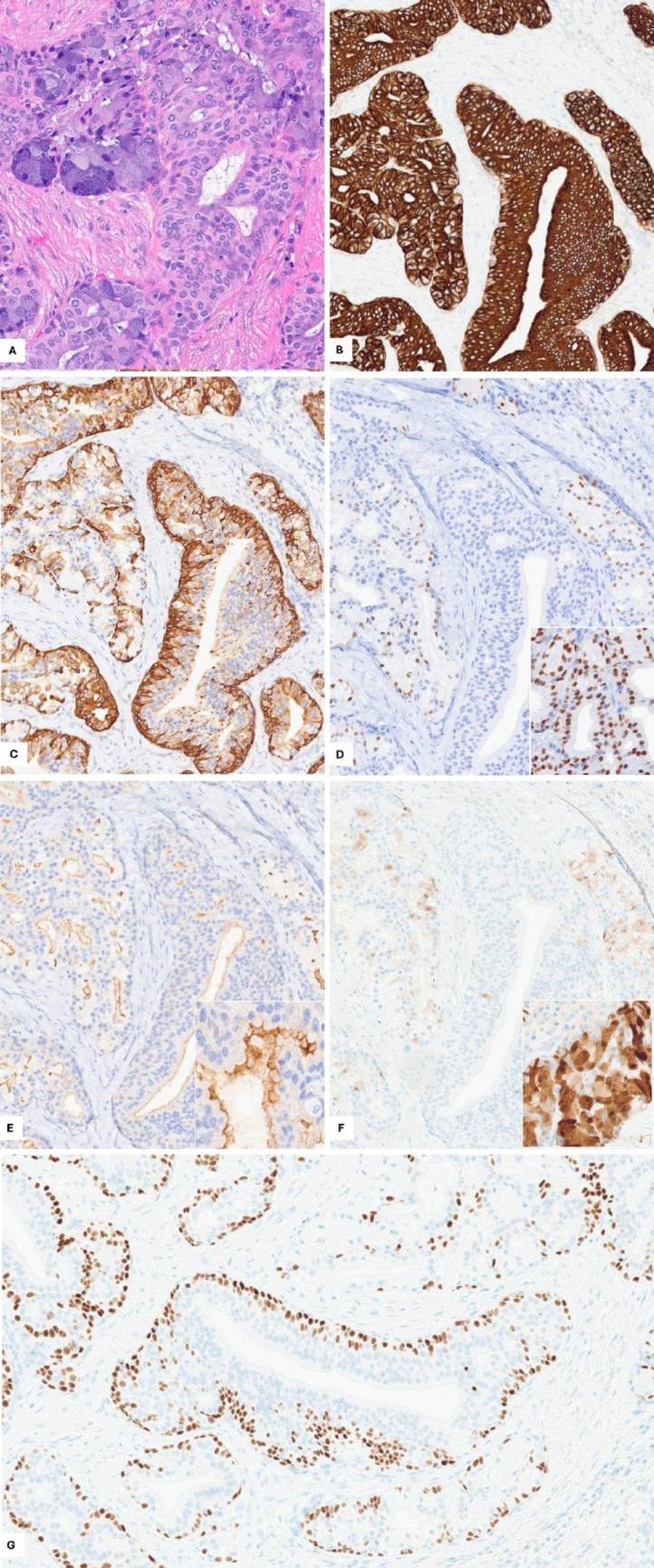
Immunohistochemical studies results (**B**–**G**) in a representative area of carcinoma (**A** H&E for reference). CK7 (**B**) is diffusely positive. CK5/6 (**C**) shows basally accentuated expression. SOX10 is variably positive in the serous acinar differentiation (**D** inset). DOG1 shows luminal expression (**E** inset). S100 (**F**) is patchy – showing no expression in the squamoglandular areas with weak-to-focally strong (inset) expression in the serous acinar/intercalated duct components. p40 (**G**) shows basal expression around the acini, with basal/parabasal (more diffuse) staining in the squamoglandular component

This case highlights that serous acinar differentiation is not restricted to acinic cell carcinomas, and it further supports the relation of mucoacinar carcinoma to the expanding phenotypic spectrum of MEC. Given the overlapping histomorphology and immunoprofile, molecular testing is mandatory to distinguish it from squamoglandular acinic cell carcinoma. The prognostic implications of this subtype are not known, but it should be histologically graded and clinically managed as a MEC.

## Data Availability

Data sharing is not applicable to this article as no datasets were generated or analyzed during the current study.
